# High-dose ibuprofen therapy associated with esophageal ulceration after pneumonectomy in a patient with cystic fibrosis: a case report

**DOI:** 10.1186/1471-2431-4-19

**Published:** 2004-09-13

**Authors:** Jennifer E Mackey, Ran D Anbar

**Affiliations:** 1 Department of Pediatrics, University Hospital, State University of New York Upstate Medical University, Syracuse, NY, USA

**Keywords:** cystic fibrosis, esophageal ulceration, gastrointestinal bleed, ibuprofen, pneumonectomy

## Abstract

**Background:**

Lung disease in patients with cystic fibrosis is thought to develop as a result of airway inflammation, infection, and obstruction. Pulmonary therapies for cystic fibrosis that reduce airway inflammation include corticosteroids, rhDNase, antibiotics, and high-dose ibuprofen. Despite evidence that high-dose ibuprofen slows the progression of lung disease in patients with cystic fibrosis, many clinicians have chosen not to use this therapy because of concerns regarding potential side effects, especially gastrointestinal bleeding. However, studies have shown a low incidence of gastrointestinal ulceration and bleeding in patients with cystic fibrosis who have been treated with high-dose ibuprofen.

**Case presentation:**

The described case illustrates a life-threatening upper gastrointestinal bleed that may have resulted from high-dose ibuprofen therapy in a patient with CF who had undergone a pneumonectomy. Mediastinal shift post-pneumonectomy distorted the patient's esophageal anatomy and may have caused decreased esophageal motility, which led to prolonged contact of the ibuprofen with the esophagus. The concentrated effect of the ibuprofen, as well as its systemic effects, probably contributed to the occurrence of the bleed in this patient.

**Conclusions:**

This report demonstrates that gastrointestinal tract anatomical abnormalities or dysmotility may be contraindications for therapy with high-dose ibuprofen in patients with cystic fibrosis.

## Background

Airway inflammation is thought to cause much of the lung damage in patients with cystic fibrosis (CF) [1,2]. Such inflammation has been found in infants with CF, even in the absence of bacterial infections or symptomatic lung disease [3].

Recent therapies for CF lung disease have been shown to preserve lung function by decreasing airway exposure to inflammation. For example, airway clearance, antibiotics (inhaled tobramycin and oral azithromycin), rhDNase, and oral corticosteroids have been used to help decrease airway inflammation [4-6]. High-dose ibuprofen therapy also has been shown to be effective in decreasing inflammation, probably by decreasing polymorphonuclear cell influx into the lungs [7]. We present a patient with CF who developed a rare complication of high-dose ibuprofen therapy related to his pneumonectomy.

## Case presentation

The patient was a teenager with CF who suffered for several years from right lower lobe consolidation, as a result of recurrent *Pseudomonas aeruginosa *pneumonia. The consolidation was unresponsive to intensive therapy including intravenous (IV) antibiotics, chest physiotherapy, systemic steroids, and rhDNase. The patient developed recurrent severe pulmonary exacerbations necessitating IV antibiotic therapy 4 times per year between the ages of 14 and 15 years. A computerized tomography scan of the chest when the patient was 15-years-old revealed complete collapse of his right lung. A ventilation/perfusion scan revealed only 8% of his ventilation and perfusion in his right chest, while the remaining 92% was in his left chest. In addition, the left lung had begun to herniate into his right chest. It was felt that the right lung was a recurrent source of infection, which was causing serious morbidity. Therefore, the patient underwent a total right pneumonectomy that he tolerated well.

Approximately one year later, in preparation for initiating high-dose ibuprofen therapy, the patient's stool was checked for occult blood. He was found to have occasional guaiac positive stools. An upper gastrointestinal endoscopy revealed chronic esophagitis, esophageal ulcerations, and Barrett's esophagus thought to be attributable to gastroesophageal reflux. A 24-hour pH probe revealed significant gastroesophageal reflux despite anti-reflux therapy. Therefore, a fundoplication was performed in order to prevent further esophageal damage. Subsequently, it was believed that his esophageal ulcerations resolved as multiple stools were documented to be guaiac negative.

**Figure 1:**
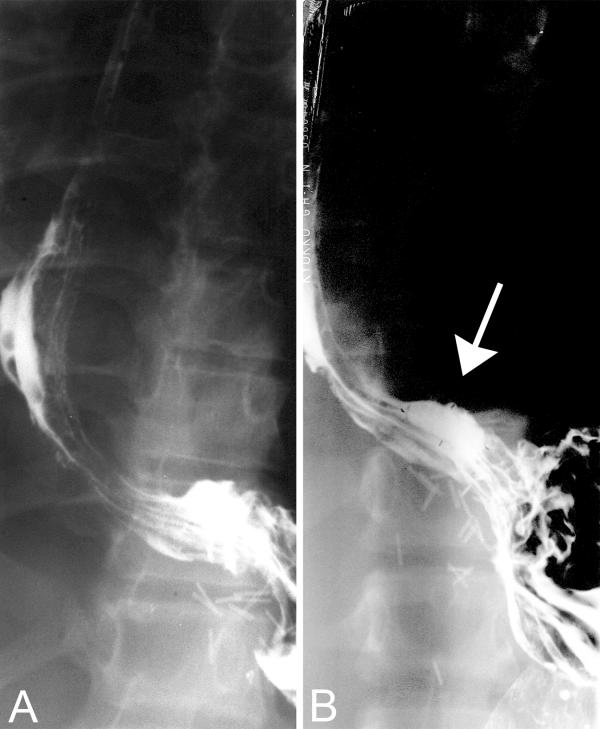
Barium swallow in a patient with cystic fibrosis following right pneumonectomy. A – The study demonstrates deviation of esophagus to the right side, and a relatively horizontal lower esophageal segment proximal to the gastro-esophageal junction. B – A pancreatic enzyme capsule emptied of the enzymes and filled with barium (indicated by the arrow) is retained within the lower esophagus for several minutes.

High-dose ibuprofen therapy was initiated when the patient was 17-years-old, based on published recommended dosage and pharmacokinetic protocols [8]. The patient's dose was determined by a pharmacokinetic analysis [8] that documented a peak ibuprofen plasma concentration of 69 mcg/ml, following a test dose of 1,000 mg (22 mg/kg). A week after initiation of ibuprofen at a dose of 1,000 mg b.i.d. the patient developed severe abdominal pain, hematemesis and bright red blood per rectum. He became hemodynamically compromised and an emergency endoscopy revealed bleeding esophageal ulcerations in the distal 12 cm of his esophagus. After stabilization and observation without further bleeding, a barium swallow demonstrated that his esophagus was deviated towards the right side and the lower segment of the esophagus was relatively horizontal proximal to the gastro-esophageal junction (Figure 1). In addition, a pancreatic enzyme capsule emptied of the enzymes and filled with barium was retained within that esophageal segment for several minutes (Figure 1).

Following this episode the patient's ibuprofen was discontinued, and his oral medications (e.g., antibiotics and vitamins) were switched to liquid form, whenever possible. His pancreatic enzymes were administered after removing the enzyme microspheres from their capsule and mixing them in applesauce.

Reported complications of pneumonectomy include mediastinal shift with herniation of the remaining lung, cardiac herniation, cardiac arrhythmias, bronchopleural fistula, esophageal motility disorders, and development of scoliosis [9-11]. It is thought that pediatric patients have more mediastinal shift following pneumonectomy than adults because of increased elasticity and compliance of the lung and mediastinum that allow for more severe anatomical derangements [9]. A study of 17 post-pneumonectomy pediatric patients revealed that all of the patients had marked herniation of the remaining lung with a mediastinal shift to the opposite side as evident on chest radiographs or computerized tomography scans [11]. One patient with a right pneumonectomy displayed excessive shift of the esophagus as well, but did not have any associated dysphagia or reflux [11]. In another study, esophageal motility was measured in 13 patients before and after pneumonectomy [9]. Patients post-pneumonectomy were shown to have esophageal dysmotility even without reporting dysphagia [9]. The dysmotility was thought to be attributable to the mediastinal shift [9]. Thus, for the patient in the present report, it is likely that the deleterious effects of gastroesophageal reflux may have been increased because of esophageal dysmotility after pneumonectomy.

In our patient, esophageal dysmotility along with the distortion of esophageal anatomy probably combined to slow the esophageal transit time of the ingested ibuprofen, which may have led to development of ulcerations as a result of prolonged concentrated contact within the esophagus. Also, the ibuprofen could have contributed to the development of ulcerations by inhibiting cyclooxygenase systemically, which decreased prostaglandin E production [12]. In turn, less prostaglandin E was available to promote bicarbonate and mucus secretion, which are protective of the gastrointestinal mucosa [12].

Delayed esophageal transit may have occurred with the patient's other oral medications prior initiation of ibuprofen. For example, his pancreatic enzymes likely were retained in the same esophageal segment, which placed him at risk of developing esophageal damage akin to the development of fibrosing colonopathy and strictures described in patients with CF who received high-dose pancreatic enzymes [13]. The enzymes may have remained inactive because the environment was not alkaline enough for their activation [14], which may be the reason that the patient did not demonstrate esophageal strictures.

According to the 2002 CF Foundation registry, only 3.8% of patients in North America with CF were treated with high-dose ibuprofen [15]. In 1999, a survey of 67 CF center physicians revealed that safety issues were a major reason that they did not prescribe this therapy [16]. Gastrointestinal bleeding, a known adverse effect of non-steroidal anti-inflammatory agents, was the safety issue of most concern to these physicians [16]. Other known side-effects of high-dose ibuprofen include renal failure (often transient), and epistaxis [3,17].

The initial randomized, double-blind, placebo control study of high-dose ibuprofen in CF did not demonstrate serious side effects during 4 years of therapy with ibuprofen sufficient to achieve peak plasma concentrations of 50–100 mcg/L [8]. Seven of 85 study patients developed abdominal pain, while only 2 of these 7 were on ibuprofen. One patient in the placebo group developed esophagitis. Of note, abdominal pain, which is very common in patients with CF, actually improved in many patients. In another randomized, double-blind, placebo controlled study, involving 19 children with cystic fibrosis; 13 children received sufficient ibuprofen for 26 months to maximum concentrations 48 +/- 17 mcg/ml, but no adverse effects could be attributed to the ibuprofen [18].

The incidence of gastrointestinal disease in patients reported to the CF Foundation registry from 1996–2000, was compared between patients who were and were not taking ibuprofen [19]. Peptic ulcer disease was reported in 0.32% of 1,186 CF patients taking high-dose ibuprofen, as compared to an incidence of 0.22% in 18,587 patients not taking ibuprofen. Gastrointestinal bleeding was reported in 0.49% of patients taking ibuprofen, as compared to 0.23% of the others (p = .0004). In the first year after initiation of high-dose ibuprofen therapy for 91 patients at the Texas Children's Hospital CF Center, one patient developed upper gastrointestinal bleeding, and one developed gastritis [20]. In a published case report, a 12-year-old patient with CF on high-dose ibuprofen developed emesis and feeding intolerance [21]. She was found to have pyloric channel stricture as a result of healing antral and pyloric ulcers, which may have been caused by ibuprofen.

## Conclusions

The risk of developing gastrointestinal side-effects from high-dose ibuprofen therapy is low for patients with CF. However, ibuprofen may be contraindicated for those who are at increased risk because of gastroesophageal reflux, history of gastrointestinal ulcerations, or abnormal gastrointestinal motility or anatomy.

## Competing interests

None declared.

## Authors' contributions

JM wrote the case report. RA treated the reported patient, and edited the report.

## Pre-publication history

The pre-publication history for this paper can be accessed here:


